# The Potential of Pectins to Modulate the Human Gut Microbiota Evaluated by In Vitro Fermentation: A Systematic Review

**DOI:** 10.3390/nu14173629

**Published:** 2022-09-02

**Authors:** Nélida Pascale, Fangjie Gu, Nadja Larsen, Lene Jespersen, Frederique Respondek

**Affiliations:** 1 CP Kelco, Cumberland Center II, 3100 Cumberland Boulevard, Suite 600, Atlanta, GA 30339, USA; 2 Department of Food Science, University of Copenhagen, Rolighedsvej 26, 1958 Frederiksberg, Denmark; 3 CP Kelco, 123 rue Jules Guesde, 92300 Levallois-Perret, France

**Keywords:** pectin, microbiota, human, prebiotic, dietary fiber, gut health

## Abstract

Pectin is a dietary fiber, and its health effects have been described extensively. Although there are limited clinical studies, there is a growing body of evidence from in vitro studies investigating the effect of pectin on human gut microbiota. This comprehensive review summarizes the findings of gut microbiota modulation in vitro as assessed by 16S rRNA gene-based technologies and elucidates the potential structure-activity relationships. Generally, pectic substrates are slowly but completely fermented, with a greater production of acetate compared with other fibers. Their fermentation, either directly or by cross-feeding interactions, results in the increased abundances of gut bacterial communities such as the family of *Ruminococcaceae*, the *Bacteroides* and *Lachnospira* genera, and species such as *Lachnospira eligens* and *Faecalibacterium prausnitzii*, where the specific stimulation of *Lachnospira* and *L. eligens* is unique to pectic substrates. Furthermore, the degree of methyl esterification, the homogalacturonan-to-rhamnogalacturonan ratio, and the molecular weight are the most influential structural factors on the gut microbiota. The latter particularly influences the growth of *Bifidobacterium* spp. The prebiotic potential of pectin targeting specific gut bacteria beneficial for human health and well-being still needs to be confirmed in humans, including the relationship between its structural features and activity.

## 1. Introduction

**Figure 1:**
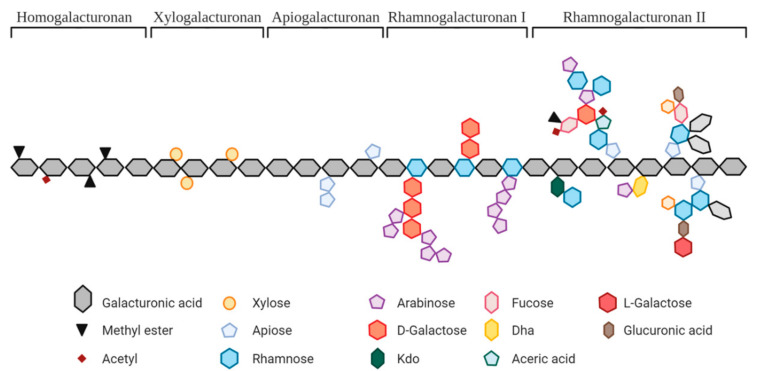
Overview of the pectin structure [3]. Copyright: Creative Commons—Attribution 4.0 International—CC BY 4.0.

Pectin constitutes a family of complex heteropolysaccharides present in the primary cell walls and middle lamella of higher plants (Figure 1). Pectin is widely used as a food additive, and its chemical structure depends on its botanical origin, part of the plant used for extraction, and extraction method. Structurally, pectin polysaccharides share some similar features. Homogalacturonan (HG) or ‘*smooth region*’ is the most abundant domain (approx. 60%) which is primarily composed of a homopolymer of partially methyl-esterified α-1,4-D-galacturonic acid (GalA) units. Rhamnogalacturonan I (RGI) or ‘*hairy*’ region consists of a backbone of repeating disaccharides based on GalA and rhamnose units. RGI regions account for about 15–20% of pectin molecules and are highly branched structures with neutral sugars (mainly arabinose and galactose) and side chains (arabinans, galactans and arabinogalactans) attached to the rhamnose units. RGI is highly present in carrots, okra, tomatoes, and potatoes, where its side chains principally contain arabinan in apples and sugar beet, and galactan in potatoes. Other pectin domains include rhamnogalacturonan II (RGII) and xylogalacturonan, the former being an overly complex branched structure composed of a HG backbone branched with L-rhamnose, D-galactose and other minor sugars. RGII typically accounts for 10% of pectin, and it has been described as the most complex polysaccharide known [1,2]. 

As a food additive, pectin on the current market is commonly obtained from citrus, apple, and sugar beet, containing a minimum of 65% of GalA. The technical classification of pectin is based on its degree of methyl esterification (DM), (i.e., the percentage of GalA units esterified with methanol). High-methoxy (HM) pectin has over 50% of its carboxyl groups esterified with methanol whereas low-methoxy (LM) pectin has less than 50% [2]. Aside from DM, other structural features govern the suitability of pectin for specific applications, including the molecular weight, GalA content, neutral sugars content, and proportion of HG:RG regions [4].

Pectin qualifies as a dietary fiber, since it is neither digested in the stomach nor the small intestine, but largely fermented in the large intestine [5]. Fiber-associated health benefits have been shown with pectin in vitro such as enhanced antihypertensive effect in fermented food products [6], as well as in vivo, such as a reduction in postprandial glycemic response and the maintenance of blood cholesterol in a normal range [7,8]. Dietary fibers can modulate the gut microbiome and specific bacterial groups, and their variety might be key to supporting it through cross-feeding interactions [9]. It could then be questioned if pectic substrates can be classified as prebiotics, a term defined as a “substrate that is selectively utilized by host microorganisms conferring a health benefit” [10]. Several human clinical studies have shown the potential benefits of pectin on gut health [11,12] (e.g., by reducing digestive symptoms such as regurgitation in infants, and the alleviation of diarrhea or intolerance symptoms in adults fed enteral nutrition) [13,14]. Overall, it is unclear how these beneficial effects are related to the fermentation of pectin in the large intestine. Until now, human clinical studies that have studied the effect of the dietary supplementation of pectin on gut microbiota composition are still scarce [15,16].

In vitro gut models have been widely used to study the impact of diet on the gut microbiota since they allow gaining insight into the fermentation processes mediated by the gut microbiota [17]. In vitro gut models vary in design, from simple batch incubations to more complex semi-continuous or multi-compartmental continuous models representing distinct parts of the human colon [18]. These fermentation models coupled with recent advances in high-throughput sequencing techniques and culture-independent methodologies, such as sequencing of the 16S rRNA gene have allowed the extensive investigation of the diversity, function, and dynamics of the gut microbial communities. Earlier (and widely employed) cultivation-independent 16S rRNA-based methods such as quantitative (real-time) PCR and fluorescent in situ hybridization (FISH) target specific bacterial groups and exhibit an overall good taxonomic resolution and sensitivity. High-throughput methods including next-generation sequencing techniques provide sequencing of PCR amplicons of the 16S rRNA gene or fragmented total (meta)genomic DNA from the whole community (e.g., Illumina, PacBio, ion semiconductor sequencing (Ion Torrent), and Nanopore sequencing, among others). Although 16S rRNA gene-based technologies pose different advantages and limitations, their adaptation allows superior monitoring of changes in the overall microbial community diversity due to fiber consumption [19].

Most of the research investigating the effects of pectin and pectin-derived substrates on the human gut microbiota has been performed in vitro, and to this date, this evidence has not been systematically reviewed [20,21]. Therefore, this review aims to be the first to systematically evaluate in vitro fermentation studies using human fecal samples to (1) determine the state of evidence of the potential effects of pectic substrates on the gut microbiota composition and their fermentative activities, and (2) clarify the potential structure-function relationships based on the complexity of the pectin molecular structure. This knowledge could aid in the better design of human clinical studies, and the development of pectin-derived ingredients with the greatest prebiotic potential.

## 2. Materials and Methods

This systematic review was conducted to elucidate the available evidence on the effects of pectin and pectin-derived substrates on the human gut microbiota using an in vitro fermentation setup. This review was conducted in line with the guidelines of the Preferred Reporting Items for Systematic Reviews and Meta-Analyses (PRISMA) statement [22].

### 2.1. Search Strategy and Eligibility Criteria

A literature search was performed in the electronic databases PubMed and Scopus on 29 January 2021. The detailed search strategies used for each database are described in Supplementary Table S1. Peer-reviewed publications were also identified by searching the reference lists of other papers, and they were identified after the search date and until submission. To be included in the systematic review, the following eligibility criteria were used: (1) an in vitro fermentation system was used with human gut microbiota as inoculum (fecal and from ileostomy), (2) pectic substrates (pectin and/or pectin-derived substrates extracted from different raw materials, and in some cases further modified by different treatments) were tested individually (not in a blend), (3) the gut microbiota composition was assessed using comprehensive molecular biological methodologies, (4) the gut microbiota composition and fermentation activity (if also studied) were included as results of the study, (5) the articles were published in the English language, and (6) the articles were published after 1 January 2010, to evaluate the most recent data available.

### 2.2. Study Screening

Two authors (F.G. and N.P.) performed the primary screening (i.e., title and abstract). The articles were assessed for their eligibility, and disagreements were resolved through consensus with a third author (F.R.). Supplementary literature searches involved examining the reference lists of all relevant studies and review articles to identify articles that were not captured in the initial search. Additional articles were selected for inclusion when deemed necessary. The full text of all potential eligible articles was retrieved, and consensus between the three investigators determined the final eligibility of each reference. Data from each eligible article were extracted by two authors (N.P. and F.R.) and included the author, subject (number, health status, and age), fecal inoculum (pooled and from a single donor), test product description and comparators included in the study, concentration of the ingredient used, pH (controlled and non-controlled), sampling time (hours and days), method of analysis of the gut microbiota, and main outcomes in terms of microbiota composition and fermentative activities.

## 3. Results

### 3.1. Study Selection and Characteristics

**Figure 2:**
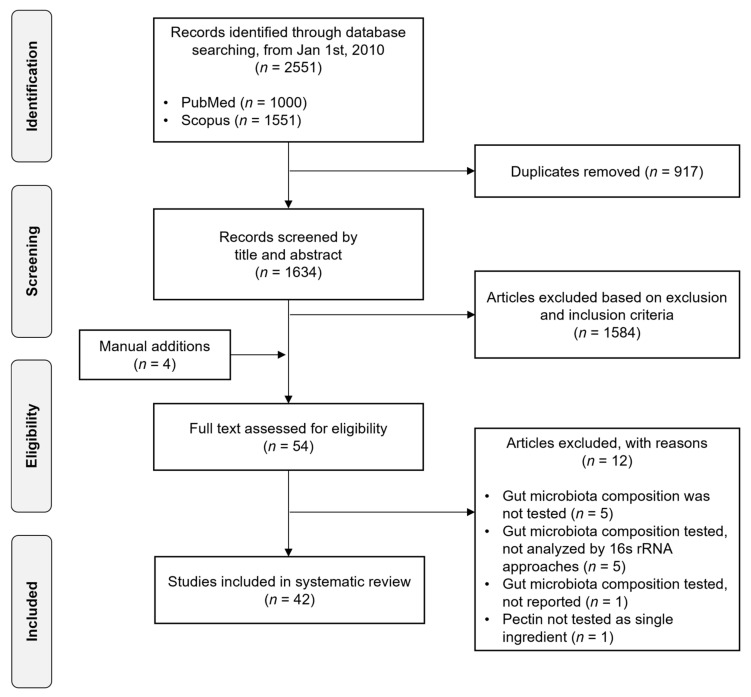
PRISMA flow diagram of studies evaluated in the systematic review.

The study identification and selection are detailed in the PRISMA flow chart (Figure 2). The initial electronic search generated 1634 unduplicated records across 2 databases. Based on the inclusion and exclusion criteria applied to the titles and abstracts of all studies, 50 publications along with 4 manually added articles were selected for a full-text review, and of those, 42 articles were included in this systematic review. 

**Figure 3:**
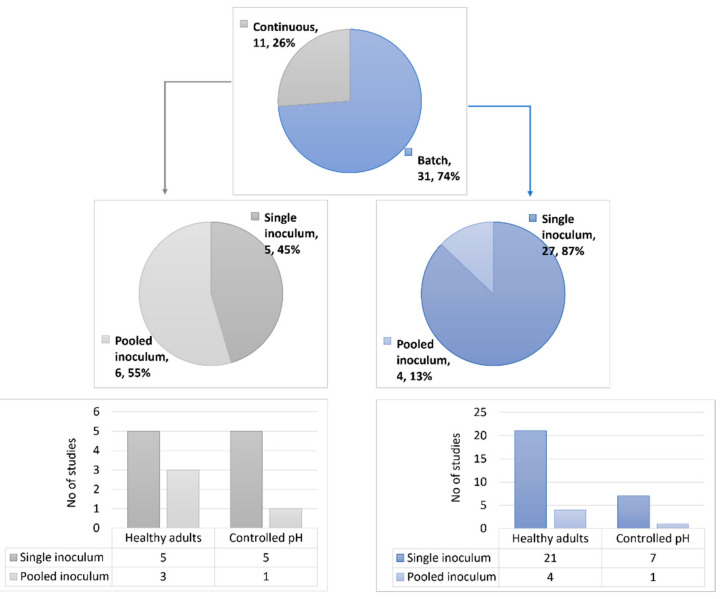
Overview of in vitro studies included in this systematic review according to their experimental design.

All 42 studies investigated the effect of pectic substrates on the human gut microbiota via in vitro fermentations, and of those, 5 studies also investigated their fermentation in upper gastrointestinal tract conditions. Overall, 31 studies were performed using batch fermentations and 11 studies used continuous fermenters, such as the Simulator of Human Intestinal Microbial Ecosystem (SHIME^®^) and the TNO in vitro colon model (TIM-2). Differences were found regarding the fecal inoculum of single donor vs. pooled, where most batch fermentations were performed using fecal samples from single donors (n = 1–17 donors). In contrast, approximately half of the continuous fermenter studies were performed with pooled fecal inocula (n = 3–8 donors) (Figure 3). Another difference found between the fermentation systems was the duration of the fermentation process. Batch fermentations were commonly performed for between 10 and 72 h, while continuous processes were mostly carried out in from 3 days to 21 days. Furthermore, pH differences were found among the batch fermentation and continuous fermenter studies, and interestingly, most batch fermentations (23 studies) were not pH-controlled. From the studies that were pH-controlled, 6 studies used a pH range resembling distal colon (DC) conditions (i.e., pH = 6.7–7.0), 1 study used a pH range of 5.8–6.3, and 1 study used 2 different conditions (i.e., pH = 5.5 and 6.5). In contrast, continuous fermenters (all pH-controlled) were either performed at a specific pH (6 studies, pH = 5.8 or 6.2) or at a pH that gradually changed during fermentation, resembling the different parts of the luminal colon (5 studies, pH = 5.6–6.9). Regarding the approach used in the technologies for analysis of the gut microbiota composition, targeted and non-targeted high-throughput technologies were used in the batch fermentation and continuous fermenter studies. Non-targeted approaches were more commonly used (18 batch fermentation studies and 9 continuous fermenter studies) than targeted approaches, and among the latter, qPCR was the most-used technology.

Thirty-three studies were conducted with fecal samples from healthy adults with a normal range for their body mass index (BMI, kg/m^2^) who did not receive antibiotics for the last 2-6 months (3 months in general). One study was performed with samples from ileostomy and not fecal samples. One study was conducted with fecal samples from the elderly. Three studies evaluated the impact of pectin on the gut microbiota from overweight or obese subjects, and two of them compared lean and obese subjects in their experiments. Two studies were conducted using fecal samples from patients with ulcerative colitis (UC), one study was conducted with fecal samples from patients (condition not defined) with no gastrointestinal disorders, and one study was conducted using fecal samples from patients with cirrhosis.

### 3.2. Tested Products

**Figure 4:**
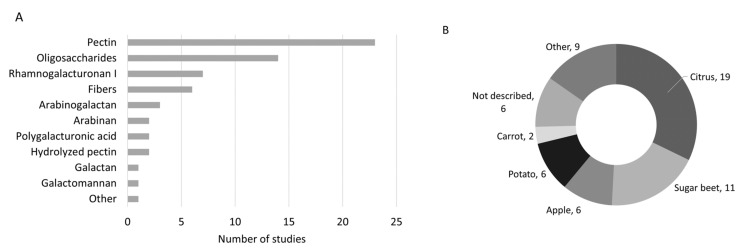
Overview of the pectic substrates tested in the studies reviewed based on (**A**) structure and (**B**) botanical origin (number of studies).

The type of pectic substrates tested varied among the studies based on raw material and structural differences (Table 1). In terms of structure, pectin was the most tested substrate, followed by oligosaccharides, RGI, and other fibers rich in pectin (Figure 4). In terms of raw material, most studies included pectic substrates from citrus and sugar beet, followed by apple and potato.

### 3.3. Effects on Gut Microbiota Composition and SCFA Production

The fermentation of pectic substrates was relatively slow (18–30 h) but complete, regardless of their structure, compared with other fibers and prebiotics. The pectic substrates were able to modulate specific populations of fecal microbiota, promoting the production of SCFA (Table 2 and Table 3). In most studies, the composition of the gut microbiota after fermentation of the pectic substrates was remarkably different compared with other prebiotics, and this specific effect was evidenced by an increased abundance at the genus, species, or even strain level.

In the phylum Bacteroidetes, the relative abundances of the genus *Bacteroides* and the *Bacteroides*-*Prevotella* group were increased in fermentations with pectin extracted from different raw materials or with polygalacturonic acid (18 studies), as well as in fermentations with RGI (4 studies). Furthermore, two studies evidenced the specific effect of pectin on the relative abundance at the species level, where increases in *Bacteroides vulgatus*, *Bacteroides stercoris* and *Bacteroides dorei* were found.

In the phylum Firmicutes, the *Ruminococcaceae* family was promoted in fermentations with native pectins (11 studies). At the genus level, *Lachnospira* was commonly increased in fermentations of pectic substrates with a variety of structures (10 studies). *Lachnospira eligens*, previously known as *Eubacterium eligens* [23], is particularly increased in pectin fermentations (four studies). In six studies, the genus *Roseburia* was promoted in fermentations with different pectic substrates, and in some of them, increased abundances of *R. intestinalis* and *R. hominis* were observed. The genus *Faecalibacterium* was commonly increased in fermentations with pectic substrates (14 studies), and of those, pectin particularly promoted an increased abundance of *F. prausnitzii* (5 studies).

A structure function relationship can be observed, and some bacteria will preferentially use a sub-part of the pectin structure (linear or branched) or a pectin with either a high or low DM. Fermentation of the pectic substrates resulted in moderate SCFA levels, with acetate being the highest and most abundant, as evidenced in 24 studies, followed by lower levels of propionate and butyrate. Furthermore, fermentation of the pectic substrates resulted in higher acetate levels (15 studies) compared with fructans. Propionate production was either low or moderate, depending on the substrate tested, and butyrate production was generally lower compared with other fibers or prebiotics and fructans in particular.

**Table 2:** Batch fermentation studies with human gut microbiota; (n = 31; 27 single donor and 4 with pooled samples (healthy vs. specific population).

Ref.	Subjects (Age, Years)	Test Products ^1^	Comparators	Methods ^2^	Main Outcomes, Including Changes in Gut Microbiota Composition and SCFA Production Linked to Pectic Substrates
SINGLE DONOR (n = 27)					
HEALTHY ADULTS (n = 21)					
*Non-controlled pH* (n = 15)					
Cantu-Jungles, 2021 [24]	10 (26–42 y)	Citrus pectin (GalA ^3^ 74%, >6.7% methoxy group, Sigma, St. Louis, MO, USA)	Blank, FOS from chicory (>95%, Sigma, USA), RS2 from potato (Bob’s Red Mill, Clackamas, OR, USA), and insoluble β-glucan	50 mg/50 mL; 0 and 24 h	β-glucan > pectin > RS2 (similar to FOS). At genus level: ↑ *Lachnospira* (negatively correlated to PRO), ↓ *Anaerostipes* (stimulated by β-glucan, negatively correlated to ACE and positively to BUT). Compared with blank, ↓ ACE in all donors (pectin > others), BUT, and PRO (β-glucan highest) than other fibers, and more various response among donors.
Wu, 2021 [25]	4 (18–30 y)	RGI-enriched fraction (MW 1.93 × 10^5^ Da, polydispersity 1.63, Rha:GalA:Gal = 1:0.8:18) from Okra fruit *(Abelmoschus esculentus,* harvested from Chengdu, Sichuan, China*)*	Basal medium, FOS (Sigma, St. Louis, MO, USA)	1% *w/v*; 0, 6, 12, 24, and 48 h	RG1-enriched fraction was partially degraded by saliva-gastrointestinal simulated conditions, and significantly fermented by the gut microbiota. At phylum level, ↑ relative Bacteroidetes and ↓ Firmicutes (compared with blank and FOS). At genus level, ↑ *Bacteroides* (14–38%), *Phascolarctobacterium, Megasphaera*, *Lachnoclostridium*, *Desulfovibrio,* and *Escherichia/Shigella* (20.6%, similar to FOS), and ↓ *Bilophila* and *Fusobacterium.* At 48 h, ↑ total SCFA (compared with blank and FOS), PRO and BUT (higher than FOS), and ↑ ACE (similar to FOS).
Yu, 2020 [26]	9 (25–40 y)	Pectin (ND)	No fiber, inulin (ND), andcellulose (ND)	5 g/L pectin, 10 g/L inulin, 20 g/L cellulose; 0 and 24 h	All subjects presented CAZymes for pectin and inulin. ↑ ACE for pectin, BUT, and H_2_ for inulin, explained by more *Lachnospiraceae* amplicon sequencing variant (ASV).
Cui, 2020 [27]	4 (age ND)	Orange or grapefruit pectin: P2 (acidic, pH 2, DE 71%), P10 (alkali, pH 10, DE 2%), C (cellulase, DE 69%), P2 + C (acid +cellulase, DE 65%), and P10 + C (alkali + cellulase, more RG1, DE 15%)	Baseline	1% *w*/*v*; 0, 4, 8, 12, 24, 48, and 72 h	Progressive fermentation over the first 48 h and stable, orange pectin > grapefruit pectin. ↑ Actinobacteria in all substrates except P2, similar abundance of Firmicutes, and ↓ Bacteroidetes; ↑*Bifidobacterium* (strongest effect) in all except P2, lowest RG1 level (26% vs. >57% in P10 and P10 + C), ↑ *Roseburia* (P10 = P2 + C > C = P10 + C), ↑ *Blautia* (P10 + C ≥ P2 + C ≥ P10 > P2) except for C, ↑ *Faecalibacterium* (P10 + C > P2 + C ≥ P10 ≥ C ≥ P2), and ↓ *Escherichia-Shigella* (P10 + C ≤ C ≤ P2 + C < P10 < P2). ↑ total SCFA, ACE (already from 4 h), PRO (from 12 h): P10 + C > P10 >> P2 + C >> C > P2. ↑ BUT: P10 + C (especially after 12 h) much higher than all other samples.
Bang, 2018 [28]	3 (29–30 y)	Citrus pectin (GalA > 74%, Sigma, St. Louis, MO, USA)	Baseline	1%; 0, 6, 12, 18, 24, 36, and 48 h	Complete degradation of pectin within 18 h. GalA produced (6–12 h) with a limited degradation within the first 6 h. Differences in substrate utilization depends on gut microbiome. ↑ *Lachnospira, Sutterella, Dorea,* and *Clostridium* (all *Clostridium* cluster XIV); ↓ *Bacteroides, Eubacterium, Rikenellaceae,* and *Roseburia.* Overall, ACE > BUT > PRO. ↑ ACE production (increased after 6 h up to 18 h, then rapidly decreased by 36 h). ↑ BUT (12–18 h and plateau at 36 h), and ↑ PRO (similar BUT trend but increased after 48 h).
Tuncil, 2017 [29]	3 donors (age ND)	PGalA from citrus pectin (Megazyme, Wicklow, Ireland)	FOS (Sigma, St. Louis, MO, USA), galactomannan (Carob) and Xyloglucan (Tamarind) (Megazyme, Wicklow, Ireland), and Arabinoxylan	50 mg/5 mL; 0, 2, 4, 6, 8, 10, 12, and 24 h	PGalA fermented rapidly overall (within 4 h), and more slowly when present in a mixture of fibers rather than alone (contrary to arabinoxylan), and 40% remained intact after 24 h. ↑ Bacteroidetes (all fibers), ↑ *Bacteroides* (all fibers), and *Lachnospira* (≈ 3 to 7–15% of relative abundance). The latter was unique to PGalA but not increased by the mixture of fibers. ↑ ACE (highest), PRO (moderate), and BUT compared with other fibers.
Min, 2015 [30]	4 (23–28 y)	High methoxy pectin (HMP, DM 76%, DP492, Tic Gums, Belcamp, MD, USA), SBP (DM 21%, DP3729, Herbstreith & Fox (Elmsford, N.Y., USA), pectin from soy (DM 23%, DP1510)	FOS (95% purity, DP 3–5, Ingredion, USA)	Unclear concentration; 0, 6, 12, 24, and 30 h	No clear effect on gut microbiota (DDGE method limitation). At 30 h: ↑ total SCFA (soy pectin > HMP = SBP > FOS), ↑ ACE (HMP = SBP = soy pectin > FOS), and ↑ BUT (FOS = soy pectin > HMP = SBP).
Van den Abbeele, 2020 [31]	1 (26 y)	RGI from carrot (min. 80% purity; Nutrileads, Wageningen, The Netherlands)	Blank and inulin (average DP > 23, Beneo, Mannheim, Germany)	5 g/L; 0, 6, 24, and 48 h; targeted bacterial groups.	Most fermentation activities between 6 and 24 h were similar between RG1 and inulin. ↑ absolute Bacteroidetes, Firmicutes, and Proteobacteria (at 48 h), similar Actinobacteria and Verrucomicrobia, within Firmicutes: ↑ *Lachnospiraceae* (lumen and mucus and 74% relative abundance in mucus, especially *Roseburia hominis*), *Ruminococcaceae (F. prausnitzii,* lumen*)*, *Veillonellaceae, Erysipelotrichaceae, Streptococcaceae, Peptostreptococcaceae,* and *Clostridiaceae* XI; within Bacteroidetes: ↑ *Bacteroidaceae* (*B. dorei, B. ovatus, B. plebeius,* and *B. xylanisolvens*), and *Prevotellaceae*; within Proteobacteria*:* ↑ *Enterobacteriaceae* and *Desulfovibrionaceae.* ↑ total SCFA, ACE, and PRO production compared with blank (mostly 6–24 h), and RGI the same as inulin. ↓ BCFA compared with blank (mostly 24–48 h): inulin < RGI < blank. LAC slightly produced between 0 and 6 h and used in 6–24 h (RGI).
Gómez, 2016 [32]	3 (age ND)	SBP, SBPOS (DM 50%, DA 37%, mostly AOS, and pH 1.8), Lemon pectin (LP), lemon POS (LPOS, DM 62%, DA 4.6%, more oligogalacturonides)	FOS from chicory (Sigma, St. Louis, MO, USA)	10 g/L; 0, 5, 10, and 24 h; targeted bacterial groups.	In the case of LPOS, fermentation began from the start for AOS and GOS, but after 5 h for OGalA and it was completed by 15–20 h. For SBPOS, fermentation of GOS and OGalA started quicker than AOS and it was complete in between 20 and 25 h. Most important with LP at 24 h: ↑ Bifidobacteria, *Lactobacillus* (similar to FOS), Bacteroidetes*, Clostridium histolyticum* cl I and II*,* and *C. coccoides/E. rectale*. Most important changes with LPOS: ↑ *Atopobium, F. prausnitzii,* and *R. intestinalis*. Generally, less bacteria increased with SBP. ↑ total SCFA, ACE (SBPOS > LPOS = FOS >> SBP > LP), and BUT (FOS >> SBPOS > LPOS > LP >> SBP).
Sulek, 2014 [33]	6 (41 ± 9 y)	Sugar beet AOS (Danisco A/S, Nakskov, Denmark), base solution (BA), LA fraction (<1 kDa), and HA fraction (>1 kDa)	No CHO in media; FOS from chicory (>95%, Beneo, Tienen, Belgium)	5 g/L; 0 and 24 h; targeted bacterial groups.	No difference in relative density of bacterial taxa between the four substrates. In comparison to control, ↑ *Bifidobacterium* spp. (1.7×), and ↓ *C. coccoides* (LA/2) but no effect on *Roseburia* spp*.,* ↓ *Alistipes* spp. (LA and FOS), or *Desulfovibrio* spp. (HA and FOS). Similar abundance of *Prevotella* spp*., Bacteroides* spp*., C. leptum* spp*., Lactobacillus* spp*., A. muciniphila,* and *Enterobacteriaceae.* LA and BA induced similar bacterial fermentation metabolites (≠ HA and FOS). Fermentation of LA and BA resulted in ↑ cysteine (pH buffer) and aminobenzoic acid. HA fermentation resulted in ↑ 3-oxoalanine, tyramine, and homoveratric acid (possibly explained by degradation of ferulic structure). AOS fermentation resulted in ↑ phenylalanine, xanthine, and linoleic acid.
Gómez, 2014 [34]	3 donors(age ND)	Orange pectin and orange POS (≈90% oligomers, 53.4% OGalA, 25.3% AOS, and 16.5% GOS)	No fiber in media; FOS (>95% purity, Sigma, St. Louis, MO, USA)	10 g/L; 0, 5, 10, and 24 h; targeted bacterial groups.	GOS were fermented first and then AOS and OGalA. Versus. control, fermentation of POS, FOS, and pectin promoted ↑ *Bifidobacterium, Bacteroides/Prevotella, Atopobium, C. coccoides/E. rectale, R. intestinalis,* and *F. prausnitzii.* POS fermentation resulted in ↑ *Lactobacillus* vs pectin (FOS intermediate). Pectin fermentation resulted in ↑ *C. histolyticum* clusters I and II vs. FOS (POS intermediate). ↑ total SCFA and ACE (POS > pectin and FOS); and ↑ PRO (pectin) and BUT (FOS).
Gullón, 2011 [35]	1 (age ND)	Apple-derived oligosaccharides: GLOS, AOS, GOS, OGalA, and XOS; total oligomers (OS)	No CHO in culture media	10 g/L; 0, 7, 10, 12, 24, 32, and 48 h; Targeted bacterial groups.	GLOS and GOS + XOS fermented first and then AOS and OGalA. ↑ *Bifidobacterium* (7–32 h), *Lactobacillus-Enterococcus* group (7–32 h), *Atopobium* cluster *(*steady increase 7–32 h), *E. rectale-C. coccoides* (10–32 h), and *C. histolyticum* (only at 32 h). Similar abundance of *Bacteroides-Prevotella.* ↑ total SCFA (LAC and SUC only detectable at 7 and 10 h, ACE increased from 2 to 32 h, and significant BUT production after 14 h). In pure cultures, all the OS (except OGalA) were partially metabolized by all *Bifidobacterium* strains (n = 5), whereas OGalA > AOS were also good carbon sources for *B. vulgatus.*
Holck, 2011 [36]	6 (41 ± 9 y)	Sugar beet AOS (Danisco A/S, Nakskov, Denmark): small (mostly DP 2–5), small and feruloylated; long (mostly DP 5–10), and long and feruloylated	FOS from chicory (>95%, DP 2–8, Beneo, Tienen, Belgium)	5 g/L; 0 and 24 h; targeted phyla (2) and genera (2)	Similar abundance of Firmicutes and ↓ Bacteroidetes in all samples; ↓ *Bifidobacterium* spp. in all samples, and in the case of long and feruloylated AOS, a similar change of this genus compared to FOS. Similar levels of *Lactobacillus* spp. *C. difficile* can grow on small AOS but not on other samples.
Thomassen 2011 [37]	3 (43 ± 10 y)	Destarched potato pulp (DNE, no enzyme), destarched potato pulp (DPP, enzyme treated), crude potato pulp (CNE, no enzyme), crude potato pulp (CPP, enzyme treated), CCP fractions: CPP < 10 kDa, CPP 10–100 kDa, and CPP > 100 kDa.	FOS from chicory (DP 2–8, Beneo, Tienen, Belgium)	5 g/L; 0 and 24 h.	CPP > 100 kDa fraction (mainly polysaccharides of HG and RGI with large galactan side chains) promoted ↑ *Bifidobacterium* (compared with FOS) and *Lactobacillus* (similar to FOS). CNE and DNE (both with low MW, <1 kDa, mainly oligosaccharides) resulted in ↑ *Bacteroidetes* (compared with FOS).
Adamberg, 2018 [9]	5 (28–48 y)	Arabino-galactan from larch tree (AG, DP > 23, Sigma, USA), and citrus pectin (GalA >74%, Sigma, St. Louis, MO, USA);	Culture medium without CHO, mucin from porcine stomach (type III, Sigma, St. Louis, MO, USA), GOS (DP 2–10, Friesland Campina, Wolvega, The Netherlands), inulin (HSI, DP 2–8), and lnulin (HP, DP > 23% (Beneo, Oreye, Belgium), Levan (DP > 100), RS (Cargill, Malchin, Germany) xylan and chitin from shrimp cells (Sigma, St. Louis, MO, USA)	5 g/L; 0, 24 and 48 h	More than 70% of all substrates were fermented by gut microbiota. Fast degradation of GOS, HSI, and RS during 24 h. Xylan and AG were fermented slowly (over 40 h). Mucin was fermented in several phases (up to 50 h) but less so compared with GOS and RS. ↑ ACE in all substrates and 80% of all SCFAs in the case of pectin. Pectin resulted in ↑ ACE and small levels of PRO and SUC, accompanied by ↑ *Bacteroides*, especially *B. vulgatus*, *B. ovatus*, *B. uniformis*, *B. faecis,* and *B. caccae*. GOS, inulins (HP and HSI), levan and RS resulted in ↑ lactate, accompanied by ↑ *Bifidobacterium* and *Lactobacillus.* Mucin, AG, and xylan resulted in ↑ PRO and SUC, along with ↑ *Bacteroides.* Mucin resulted in ↑ *Clostridium, Parabacteroides,* and *Lachnoclostridium.* Xylan and AG promoted ↑ BUT and PRO compared with all substrates.
*Specific pH range (n =6)*					
Johnson, 2015 [38]	3 (age ND)	Pectin (ND)	Control medium (low fibers and inulin (ND)	1.5 g; pH 6.7–6.9; 0, 5, 10, 24, 30, and 48 h.	Pectin more slowly fermented between 0 and 10 h compared with inulin, but a similar level at 30 h. ↑ *Bacteroides* with pectin fermentation (confirmed by targeted FISH). Most SCFA production between 0 and 10 h. Pectin (=inulin) resulted in ↑ ACE and BUT. Inulin resulted in ↑ PRO, ISOBUT, VAL, and ISOVAL compared with pectin.
Reichardt, 2018 [39]	3 (age ND)	RGI from potato (Megazyme, Bray Ireland), and apple pectin (Sigma, UK)	FOS (95%, DP 2–8) and Inulin (99%, DP > 23) (Beneo, Tienen, Belgium), arabinoxylan (Megazyme, Bray, Ireland), barley β-glucan (PolyCell Technologies, Crookston, USA), RS2 and RS3 (National Starch and Chemical Comp., Bridgewater, USA), FiberSol (Matsutani, Itami-City, Japan)	0.2% *w/v*; pH 5.5 and 6.5; 0, 6, and 24 h	↑ *F. prausnitzii* with apple pectin (both pH levels, stronger at pH 5.5). At pH 6.5 only: ↑ *E. hallii* (apple pectin > RGI > fructans). RGI: ↑ *L. eligens* ^4^ (more strongly at pH 6.5, 4× vs. control), compared with apple pectin. ↑ *Bacteroides* spp. (at pH 6.5, 2.6×) compared with same levels with β -glucans and apple pectin. Apple pectin (and fructans): ↑ *B. longum* (at pH 5.5) which was higher vs. RGI and RS. ↑ total SCFA measured after 24 h, similar range to other NDCs, mostly ACE, and compared with BUT for fructans.
Di, 2017 [40]	5 (30 ± 7 y)	POS1 (MW 72.8 × 10^3^, DM 40%, Gal:Rha 3.14), POS2 (MW 811 × 10^3^, DM 42%, Gal:Rha 1.97), MCP1 (MW 9.2 × 10^3^, DM 5%, Gal:Rha 2.92), and MCP2 (MW 17.7 × 10^3^, Gal:Rha 4.47, DM 3%) from orange peels (EcoNugenics Inc., Santa Rosa, CA, USA)	Inulin (99%, Beneo, Tienen, Belgium)	1% *w/v*; pH 6.7–6.9;0, 10, 24, 36, and 48 h; targeted bacterial groups.	↑ *Bifidobacterium*: Inulin > POS2 and MCP1 (latter similar to POS1). ↑ *Lactobacillus/Enterococcus* from fermentation of inulin (similar abundance with POS2). Lower levels were found with MCP1 and POS1. Similar levels of *E. rectale/C. coccoides* (and no SD found) among substrates. Abundance of *Bacteroides/Prevotella* and *Atopobium* clusters was higher, but no SD were found among substrates. MCP1 promoted ↑ *C. histolyticum* (36 h only). ↑ total SCFA, ACE, and PRO: already after 10 h, plateau at 36 h, and no SD among substrates; ↑ BUT (after 24 h): inulin > POS2 > POS1 = MCP1. Anti-adhesive activity against *E. coli* O157:H7 strongest with POS1, MCP1, and MCP2 (only tested for that). Mechanism of action for POS1 possibly linked to high GalA:Rha ratio, and intermediate DM and MW. For low MW, de-esterified structures enhanced STEC anti-adhesive activity.
Moon, 2015 [41]	3 (age ND)	Debranched sugar beet arabinan (LAR, average MW 18 kDa, Megazyme, Wicklow, Ireland) and sugar beet linear AOS (LAOS, 50% DP3, 29% DP2, 20% DP4, and 1% DP5).	FOS (DP 3–5, Wako, Osaka, Japan)	1% *w/v*; pH 6.8; 0, 12, and 24 h; targeted bacterial groups.	LAOS and LA: slower fermentation than FOS and less rapid ↑ *Bifidobacterium* with LAOS but similar to FOS at 24 h. No effect of LAR on tested bacterial groups (slight ↑ *Bacteroides* but not significant). ↑ LAC: FOS >> LAOS > LAR; ↑ total SCFA; ↑ ACE: FOS = LAOS > LAR; ↑ PRO: LAR > LAOS > FOS, and ↑ BUT: FOS = LAOS = LAR.
Onumpai, 2011 [42]	4 (30 ± 4 y)	PGalA (Sigma, St. Louis, MO, USA); OGalA DP5 (DP 1–10), OGalA DP 9 (DP 4–23), methylated citrus pectin (MPec, DM 34.5%, Danisco A/S, Copenhagen, Denmark), methylated OGalA (MOGalA, DP 1–10), RGI (*A. thaliana* seed mucilage), oligorhamnogalacturonides (Orham, DP 2–19), potato galactan and beet arabinan (British Sugar, Peterborough, UK), oligogalactosides (PGOS, DP 1–10), oligoarabinosides (OAr, DP 1–11)	Inulin (>97%, Beneo ST, Orafti, Tienen, Belgium)	1% *w/v*; pH 6.7–6.9;0, 12, 24, and 36 h; targeted bacterial groups.	In comparison to probe (t = 0 h): ↑ *Bifidobacterium* (only with arabinan, OAr, galactan, PGOS and inulin), *Lactobacillus/Enterococcus* (similar abundance except ↑ with inulin), *E. rectale/C. coccoides* (similar abundance except ↑ with OAr at 36 h), *C. histolyticum,* and the *Bacteroides-Prevotella* group (less efficient OGalA DP5, Orham, OAr, galactan and PGOS), and ↓ *F. prausnitzii* but higher level with MOGalA, MPec, and arabinan. ↑ SCFA: PGOS ≥ galactan = arabinan = MOGalA ≥ OAr = Orham > all others, ↑ ACE: MOGalA = PGOS ≥ galactan ≥ most samples > inulin OGalA DP9, ↑ PRO: Orham = arabinan > most samples > MPec, ↑ BUT: inulin ≥ PGOS = galactan ≥ most samples > MPec = OGalA DP5, ↑ LAC: only with arabinan, OAr, galactan, PGOS, and inulin (12–24 h).
Ferreira-Lazarte, 2018 [43]	5 (31± 4 y)	Sunflower pectin (DM 45.7%, 800–100 kDa), sunflower MP (DM 17%, 12.5 kDa), Artichoke pectin (DM 8.9%, >500 kDa), artichoke MP (DM 8.5%), citrus pectin (Ceamsa, Pontevedra, Spain, DM 70.7%), and citrus MP (DM 14.2%)	Negative: no CHO. Positive: FOS (ND) and inulin (ND).	1% *w/v*; pH 6.7–6.9;0, 10, 24, 36, and 48 h; targeted bacterial groups.	↑ *Bifidobacterium* for all substrates >24 h and highest for artichoke MP. Low MW correlated with ↑ *Bifidobacterium* for citrus and artichoke MP vs. parent pectins, while DM had no impact. ↑ *Bacteroides/ Prevotella* for INU, FOS, and artichoke MP, with highest growth for MP (sunflower and artichoke) vs. parent pectins. ↑ *Lactobacillus /Enterococcus* for all substrates (highest with INU and artichoke MP) and low MW correlated with ↑ *Lactobacillus/Enterococcus* for citrus and artichoke but not sunflower samples. ↑ *C. coccoides/E. rectale* for all substrates, but no differences in growth due to MW variation. ↓ *C. histolyticum* for all substrates. ↑ total SCFAs in all substrates in 10–24 h. No impact from MW or DM. ACE > PRO > BUT for all substrates. SD in ACE found only between artichoke and citrus MP. ↑ PRO (for all substrates at 48 h, highest INU, FOS), and BUT (for all substrates at 24 h and highest INU and FOS at 48 h).
SPECIFIC POPULATIONS (n = 6)					
*Non-controlled pH (n = 5)*					
Van Trijp, 2020 [44]	5 ileostomy subjects (30–75 y)	Lemon pectin (DM 67%, CP Kelco, Lille Skensved, Denmark)	Inulin and FOS (DP 2–60, Sensus, Roosendaal, the Netherlands), GOS (69%, DP 2–6, Friesland Campina, Wageningen, the Netherlands), and potato IMMP (92% α-1-6, average DP 50, Avebe, Veendam, Belgium)	10 g/L; 0, 5 ,7 ,9, and 24 h	Seven hour lag time for pectin (slow fermentation), thus tested in only 2 subjects (28 and 20 g/day fiber). ↑ *Cellulosilyticum* (1 subject, between 9 and 24 h), otherwise, no modification. Most of SCFA produced between 9 and 24 h (contrary to FOS and GOS); ↑ ACE and ↓ PRO.
Yang, 2013 [45]	15 adult patients (age ND)	Pectin (TIC gums, White Marsh, MD, USA): 35% polymeric uronic acid residues, DM 72%, MW peak at 9.4 × 10^5^, and 38% free glucose; botanical origin ND	Guar gum (TIC gums, White Marsh, MD, USA), agave inulin (Ciranda, Hudson, WI, USA), corn RS2 (70% high amylose, Cargill, Cedar Rapids, IA, USA), oat β-glucan (Quaker, Chicago, IL, USA), corn arabinoxylan (AX, Bunge Milling, Danville, IL, USA)	1% *w/v*; 0 and 12 h.	↑ Actinobacteria (highest with pectin) and Proteobacteria, ↓ Bacteroidetes and Firmicutes (greatest with pectin), ↑ *Bifidobacterium* (greatest with pectin) and *Collinsella* (inulin > pectin > others), and ↓ *Blautia* (RS2 < guar gum < pectin < others) and *Bacteroides* (pectin ≤ inulin < others). ↑ total SCFA, ACE (pectin similar to all others), PRO (pectin ≤ inulin < AX = β-glucan ≤ RS2 ≤ guar gum), and BUT (RS2 ≤ pectin = AX ≤ β-glucan ≤ guar gum ≤ inulin).
Vigsnæs, 2011 [46]	12 UC patients with 6 healthy adults (41 ± 9 y)	Sugar beet AOS (DP 2–10, Danisco A/S, Nakskov, Denmark) and arabinose moiety (85 mol%, 125 mg/g free sugars, ferulic acid 36 µg/g)	No substrate; FOS (95%, Beneo, DP 2–8, Tienen, Belgium)	5 g/L; 0 and 24 h; targeted bacterial groups.	Less Bacteroidetes and *F. prausnitzii* (not significant) in UC vs. healthy subjects. ↓ Bacteroidetes (both healthy and UC, =FOS) and Firmicutes (both healthy and UC, < FOS), ↑ *Bifidobacterium* (only in UC relapse = FOS), and *Lactobacillus* (remission and relapse = FOS). ↓ *C. coccoides* and *C. leptum* groups, and *Desulfovibrio* spp. (might be pH related; both healthy and UC = FOS). Versus control (no substrate), pH decreased from 6.5–7.5 to 5.5–6 during AOS fermentation. Similar total SCFA, PRO, BUT; ↑ ACE (only in relapse UC, =FOS).
Holck, 2011 [47]	3 UC remission (36 ± 5 y); 3 UC relapse (44 ± 6 y); 3 healthy (43 ± 10 y)	HG oligosaccharides (DP4 and DP5) from SBP (Danisco A/S, Nakskov, Denmark)	Baseline	5 g/L; 0 and 24 h; targeted phyla (n = 2).	No difference between healthy and UC patients. ↑ Firmicutes: DP4 only; ↓ Bacteroidetes: DP4 <DP5 < inoculum.
Jin, 2019 [48]	17 patients with cirrhosis and 17 healthy (18–80 y)	Citrus pectin (Unipectine™, Cargill Inc., Wayzata, MN, USA)	Baseline, RS type 4 (Fibersym^®^ RW, MGP Ingredients, Atchison, KS, USA), lactulose (LL, Sigma, St. Louis, MO, USA), arabinoxylan (AX, Corn Biofiber Gum Agrifiber Holdings LLC, (Mundelein, IL, USA)	2%; 0 and 14 h	Cirrhosis affected capacity to produce SCFA from pectin fermentation. ↑ unclassified *Ruminococcaceae, Faecalibacterium,* and *Ruminococcus.* ACE > PRO > BUT in both patients and controls. Pectin effect in cirrhotic patients: ↓ total SCFA, ACE, and BUT, and similar PRO. Pectin effect in healthy controls: ↑ BUT.
*Specific pH range (n = 1)*					
Adamberg, 2018 [49]	7 OW (7–14 y) with 6 healthy NW (4–15 y)	Apple pectin (AP, Sigma, St. Louis, MO, USA)	Arabinogalactan (AG)	From 0.2 L/h to 0.06 L/h; pH 7; 0 and 10 h	Lower growing rate with apple pectin. Most changes observed at species level (≤10% of initial population) but no clear difference between the carbohydrates. No clear substrate specificity. ↑ *B. vulgatus* (fast growing bacteria) in NW only. ↑ ACE in both OW and NW with AP.
POOLED (ALL HEALTHY DONORS) (n = 4)					
*Non-controlled pH (n = 3)*					
Perez-Burillo, 2019 [50]	3 (mean BMI 21.3, age ND)	Citrus fiber (42% pectin and 25% cellulose and hemicellulose; Fiberstars, USA)	Control salami (no fiber), inulin (99.5%, Beneo, Belgium), acacia gum (Arabinogalactan, Nexira, France)	2% in salami; 0 and 24 h	↑ *Dorea* and *Clostridium* cluster XIVb with citrus and acacia fiber. ↓ *Escherichia/Shigella* with citrus and acacia fiber.↑ SCFA (total and individuals) vs. control salami for all fiber-salami.
Cantu-Jungles, 2019 [51]	3 (age ND*)*	Isolated highly-branched RGI (AGI), HG, and AGI (uronic acid/(Ara + Gal): 1.3, HG − DM 79%). Xyloglucan (XYG, tucumã pulp).	FOS (95%, Sigma, St. Louis, MO, USA)	1% *w/v*; 0, 4, 8, 12, and 24 h	Slower fermentation of XYG compared with pectic substrates and FOS. At 12 h, ↑ Bacteroidetes, ↓ Firmicutes (especially AGI), ↑ *Bacteroides*, *B. plebeius* (AGI > HG + AGI > FOS), and *Lachnospira* (HG + AGI (4.1%) > AGI (0.6%) = FOS). ↑ Gas production found: FOS > HG + AGI > AGI; ↑ total SCFA (=FOS), ACE (HG + AGI > AGI = FOS), PRO (AGI > HG + AGI = FOS), and BUT (FOS >> AGI/HG + AGI).
Leijdekkers, 2014 [52]	10 (44 ± 7 y)	SBPOS (90%, 15% average DP5, GalA 43%; Cosun, Breda, the Netherlands)	FOS (95%, Sensus, Roosendaal, the Netherlands)	1% *w/v*; 0, 3, 6, 9, 12, and 24 h	Slower fermentation than FOS (0–12 h). ↑ *Blautia, Lachnospira_incertae_sedis, Faecalibacterium (*compared with FOS after 9 h), *Parabacteroides, Paraprevotella,* and *Bifidobacterium (*most fermentation between 3 and 12 h, slower than FOS). ↑ total SCFA (1.5× vs FOS), ACE (2.5× vs FOS), PRO (5.6× vs FOS), and BUT (0.6× vs FOS). No lactate (<FOS) and ↓ pH (remains > FOS).
*Specific pH range (n = 1)*					
Ramasamy, 2014 [53]	8 (25–45 y)	Chicory root pulp (62% pectin and 38% uronic acid; Sensus, Roosendaal, the Netherlands)	Baseline	1% *w/v*; pH 5.8–6.3;0, 2, 6, 8, 12, and 24 h.	24–31% of CHO from substrate not fermented after 24 h (mostly from insoluble fraction, with pectin being in soluble fraction). At 24 h only (% abundance): ↑ *Bacteroides, Clostridium sensu stricto* sp. and *Lachnospiraceae incertae sedis*, and ↓ *Sutterella, Dorea, Clostridium* cluster XIVa, and *Faecalibacterium.* ↑ total SCFA, progressive after 6 h.

Legend: Upward arrows indicate an increased bacterial abundance, and downward arrows indicate a decreased bacterial abundance. ^1^ Test products were described as in the studies. They were produced under laboratory conditions when no supplier is mentioned. ^2^ Concentration, sampling time, and microbiota determination. All studies used a non-targeted bacterial group determination approach within their methodology unless specified. ^3^ ACE: acetic acid; Ara: arabinan; AOS: arabino-oligosaccharides; BUT: butyric acid; CHO: carbohydrates; DE: degree of esterification; DM: degree of methylation; DP; degree of polymerization; FOS: fructo-oligosaccharides; Gal: galactose; GalA: galacturonic acid; GOS: galacto-oligosaccharides; GLOS: gluco-oligosaccharides; HG: homogalacturonan; IMMP: isomalto/malto-polysaccharides; ISOBUT: isobutyric acid; ISOVAL: isovaleric acid; LAC: lactate; MW: molecular weight; MP: modified pectin; OGalA: oligogalacturonides; PGalA: polygalacturonic acid; POS: pectic oligosaccharides; PRO: propionic acid; Rha: rhamnose; RG: rhamnogalacturonan; RS: resistant starch; SBP: sugar beet pectin; SCFA: short-chain fatty acids; SBPOS: sugar beet pectic oligosaccharides; SD: significant differences; SUC: succinate; UC: ulcerative colitis; VAL: valeric acid; XOS: xylo-oligosaccharides. ^4^ *Lachnospira eligens* previously known as *Eubacterium eligens* [23].

**Table 3:** In vitro continuous fermenter studies with human gut microbiota; n = 11: 5 single donor; 6 with pooled samples (healthy vs. specific population).

Ref	Subjects (Age, Years)	Product Tested ^1^	Comparator	Methods ^2^	Main Outcomes, Including Changes in Gut Microbiota Composition and SCFA Production Linked to Pectic Substrates
SINGLE DONOR (ALL HEALTHY DONORS) (n = 5)					
Chung, 2019 [54]	2 (53–64 y)	Apple pectin (Unipectin, Cargill, Belgium)	Inulin, AXOS ^3^, mixture 1 (all), and mixture 2 (all and RS, galactomannan, and β-glucan)	4.2 g/L (for single substrate); pH 6.1 ± 0.1; 20 days; single-stage anaerobic	↑ *B. vulgatus* (13.4%), *B. stercoris* (4.8%), *L. eligens* ^4^ (0.93%), unidentified *Ruminococcaceae* (0.41%), *F. prausnitzii *(0.40%), *Ruminococcus* sp. (0.22%), *Roseburia* sp. (0.19%), and unclassified *Lachnospiraceae* (0.16%). ↑ ACE and ↓ PRO compared with inulin. *B. vulgatus* has 44 CAZymes to degrade pectin, 55 *B. dorei* and 17 *B. stercoris* (hydrolase, lyase, and esterase activities).
Chung, 2016 [55]	3 (age ND)	Apple pectin (Sigma, St. Louis, MO, USA)	Inulin DP < 10 (Oligo-Fiber DS2, Cargill)	0.5% *w/v*; pH 5.5–6.9; 12 days; single-stage anaerobic	Different and complementary effects of pectin and inulin. Pectin’s most abundant species: *B. vulgatus/dorei* (17%, more neutral pH), *L. eligens* (15%, no clear effect of pH), *B. stercoris* (7%), and *F. prausnitzii* moderately increased especially at lower pH (same extent as inulin). Pectin resulted in ↑ ACE and inulin in ↑ BUT.
Ferreira, 2019 [4]	1 (age ND)	Citrus pectin, (DM 70%, average MW 350 kDa, GalA 66%, Ceamsa, Pontevedra, Spain)	Baseline	3% *w/v*; pH 5.6 (AC), 6.3 (TC), and 6.8 (DC); 14 days; SIMGI; targeted bacterial groups	High resistance of pectin to upper gastrointestinal digestion (no changes in stomach and slight decrease in small intestine), and degradation starts in AC. ↑ *Bifidobacterium* (+3 log), *F. prausnitzii* (+2 log), *Bacteroides* spp. (+1 log in AC, TC), *Enterobacteriaceae* (AC and DC) and *Enterococcaceae* (AC only), and ↓ *Lactobacillus* (AC, TC, and DC). ↑ total SCFA (AC, TC and DC): ACE > PRO > BUT; ↑ ACE (AC, TC, and DC), and BUT (mostly TC and DC), similar PRO, LAC, and BCFA, and ↓ NH_4_^+^.
Van den Abbeele, 2021 [56]	4 (29–33 y)	RGI (80%, carrot, Nutrileads, Wageningen, the Netherlands)	Baseline	3 g/d;pH 5.7–5.9 (PC) and 6.6–6.9 (DC);21 days;SHIME^®^	↑ *Bacteroidaceae* (PC, DC lumen, and PC mucus), *Prevotellaceae* (lumen and mucus), *Lachnospiraceae* (lumen), *Ruminococcaceae* (PC lumen), and *Xanthomonadaceae* (DC lumen); ↓ *Ruminococcaceae* (DC), *Veillonellaceae* (PC mucus), and *Desulfovibrionaceae*. Among top 25 OTUs: ↑ *Prevotella* sp., *B. longum*, *B. dorei* (lumen), *Phascolarctobacterium faecium*, *Lachnoclostridium* sp. (PC lumen), and *C. clostridioforme bolteae* (DC lumen) and ↓ *B. bifidum*, *B. massiliensis*, *Cloacibacillus* sp. (DC lumen), and *C. clostridioforme/bolteae* (PC mucus). ↑ ACE and PRO dominated SCFA (PC and DC), ↑ BUT (PC and DC), ↓ BCFA (both PC and DC) and no change in NH_4_^+^.
Khodaei, 2016 [57]	1 (age ND)	RGI from potato (90.8% polysaccharides, 6.5% DP 2–70, and 2.7% DP1; Megazyme, Wicklow, Ireland); oligo-RGI (51% DP 2–12 and 6.3% DP1; 73% Gal), Oligo-RGI (GOS, no polysaccharides, 51% DP 13–70, and 6.1% DP1; 70% Gal).	FOS (>95%, DP 2–8, Beneo, Belgium); 3.2 g/L CHO as negative control	9.7 g/L; pH 6.2; 4 days; BIOSTAT^®^; targeted bacterial groups	No difference found between the 2 types of oligosaccharides: ↑ *Bifidobacterium* (RGI < both OS = FOS), *Lactobacillus* spp. (moderate), *Enterococcus* spp. (vs control, both OS > RGI = FOS), *Enterobacteriaceae* (RGI > OS = FOS), and *B. coccoides* (all samples). ↓ *Clostridium leptum* (RGI< both OS = FOS). Similar abundance of *Bacteroides, Prevotella, and Porphiromonas*. ↑ total SCFA: OS ≥ RGI = FOS; ↑ ACE: OS ≥ RGI = FOS; ↑ PRO: RGI > OS = FOS; ↑ BUT: FOS > RGI = OS.
POOLED (n = 6)					
HEALTHY ADULTS (n = 3)					
Larsen, 2019 [58]	8 (25–42 y)	Potato fiber (FiberBind, KMC, Brande, Denmark, 65% dietary fiber, containing pectin, cellulose, and hemicellulose). Pectin fraction consisted of GalA (13.1%) and rhamnose (0.5%)	Baseline, native potato starch (NS), and potato cross-linked resistant starch (RS)	7.5 g/d; pH 5.8; 0, 24, 48, 56, and 72 h TIM-2	Compared with baseline when different from starches: ↓ *Lachnospiraceae* (>NS), ↑ *Clostridiaceae* (<NS), *Mogibacteriaceae* (>NS), *Christensenellaceae* (<NS), *Enterobacteriaceae* (<NS), and *Desulfovibrionaceae* (NS); ↑ *Lachnospira* (0.32–2.29%, >NS and RS), *Butyrivibrio* (0.04–0.41%, >NS and RS), and *Mogibacterium* (<0.01–0.65%, >NS and RS); ↓ *Prevotella* (2.25–17.0%; >NS, RS), and *Bacteroides* (1.64–4.18%, >NS); ↑ *Bifidobacterium* (0.08–0.25%, <NS), ↓ *Blautia* (>RS) and *Ruminoccocus*_Other (<NS and RS); ↓ *P. copri* (15.8–1.13%, <RS -> NS), *R. gnavus* (>RS), and *R. torques* (>NS and RS); ↑ *B. uniformis* (<RS), *B. ovatus* (> NS, and RS), *P. distasonis* (<RS), and *Desulfovibrio D168* (>NS and RS). No difference in total SCFA vs. starches, and ACE > PRO > BUT for all substrates.
Larsen, 2019 [59]	8 (25–42 y)	Citrus pectins with various DM and extraction processes (P1–P3, P5–P8; CP Kelco, Lille Skensved, Denmark), SBP (P4), and RGI (P10).	Baseline	7.5 g/d; pH 5.8; 0, 24, 48, 56, and 72 h; TIM-2	Common to all pectic substrates: ↑ *Ruminococcaceae* (except lime RGI); ↓ *Lachnospiraceae* and *Erysipelotrichaceae;* ↑ *Oscillospira, Mogibacterium citrobacter, Lachnospira, Prevotella* (except lime RGI), and *Butyrivibrio* (except lime RGI and SBP); ↓ *Ruminococcus*, *Roseburia,* and *Catenibacterium.* ↑ total SCFA and ACE. Based on substrate differences: GalA positively correlated with *Ruminococcaceae* and *F. prausnitzii.* Arabinose positively correlated with *P. copri.* DM positively correlated with *Ruminococcaceae,* and species *F. prausnitzii* and *P. copri.* DB positively correlated with *Lachnospira* and *Coprococcus.* For lemon pectin, no impact from mild, harsh, or “other” extraction type. ↑ PRO (highest with SBP and RGI) and BUT (highest with orange pectin non-amidated and lowest with RGI). PRO positively correlated with *P. copri* and negatively with *Lachnospira.* BUT negatively correlated with *Bacteroides.*
Bianchi, 2019 [60]	3 (age ND)	Lemon pectin (harshly extracted, LM, CP Kelco, Lille Skensved, Denmark) and probiotic strain *B. longum* BB-46 (Chr. Hansen, Hørsholm, Denmark)	Probiotic strain only	2% *w/v* (8 g/d); pH 5.6–5.9 (AC), 6.1–6.9 (TC), and 6.6–6.9 (DC); 7 days for each treatment; SHIME^®^.	Low richness and diversity with pectin + probiotic treatment. ↑ *Lactobacillaceae* (0.28–37.51%) and *Enterobacteriaceae* (16–33%) in AC. ↑ *Ruminococcaceae* (3–73% in TC, and 10–43% in DC). ↑ *Veillonella, Lactobacillus, Enterobacter, Klebsiella,* and *Erwinia* in AC. ↑ *Faecalibacterium, Eubacterium,* and unclassified *Ruminococcaceae* in TC and DC. ↓ *Enterobacter, Klebsiella, Erwinia, Bacteroides, Peptinophilus,* and *Streptococcus* in TC and DC. ↑ ACE, BUT, and PRO (TC and DC). ↓ NH_4_^+^ (compared with control and probiotic alone, positively correlated to *Streptococcus, Bacteroides, Clostridium* and *Peptinophilus*).
SPECIFIC POPULATIONS (n = 3)					
Aguirre, 2014 [17]	4 lean healthy adults (BMI 23) and 4 obese adults (BMI 33) (age ND)	Apple fiber (23% uronic acid; CSM, Bingen, Germany) and SBP (GENU pectin, DE 53%, and 58% uronic acid; CP Kelco, CPKelco, Nijmegen, the Netherlands).	SIEM (control), GOS (97%, DP 2–6; Friesland Campina, Beilen, the Netherlands), lactulose (98%, Sigma, Zwijndrecht, the Netherlands)	7.5 g/d; pH 5.8; 0 and 72 h; TIM-2 (PC conditions)	Apple fiber and SBP poorly fermented *vs* GOS and lactulose in all experiments (lean and obese). In comparison with SIEM, Firmicutes: ↑ *Catenibacterium* (215× apple in lean), *Anaerostipes* (46× apple in lean, 27× apple in obese = lactulose), *Clostridium* cluster XIVb (21× SB in lean), *Faecalibacterium* (6× SB in lean, 8× SB in obese but << GOS and lactulose in obese), *Dorea* (46× apple in obese = GOS = lactulose), and *Lachnospiraceae incertae sedis* (23× apple in obese). Similar abundance of *Blautia* in obese contrary to GOS and lactulose, which promoted an increased abundance of *Blautia*. Bacteroidetes: ↑ *Parabacteroides* (6× apple in lean and SB in obese), *Bacteroides* spp. (22× SB lean); Actinobacteria: ↑ *Bifidobacterium* (12× SB in lean but << GOS and lactulose); Proteobacteria: ↑ *Parasutterella* (10× apple and SB in lean > GOS and <lactulose). ↑ ACE: GOS = lactulose > SB pectin > apple fiber; ↑ PRO: SB pectin > apple fiber > GOS = lactulose; ↑ BUT: GOS > lactulose = apple fiber > SB pectin; ↑ total SCFA for apple fiber and SB pectin: lean > obese.
Bianchi, 2018 [61]	3 obese adults (BMI > 30 Kg/m^2^, age ND)	Lemon pectin (harshly extracted, DM 36%, CP Kelco, Lille Skensved, Denmark)	Baseline	2% (*w/v*); pH 5.6–5.9 (AC), 6.1–6.4 (TC), 6.6–6.9 (DC); 7 days; SHIME^®^	↑ Firmicutes and Proteobacteria, ↓ Bacteroidetes; ↑ *Ruminococcaceae, Succinivibrionaceae,* and *Alteromonadaceae,* ↓ *Lachnospiraceae,* (20–25% in AC and <10% in TC) and ↓ *Clostridium* spp., *Bacteroides,* and *Streptococcus.* Lower diversity because genera *Ruminococcaceae_unclassified* represented 33–53% and *Succinivibrio* represented 28–37% of gut microbiota. ↑ total SCFA in all parts of the colon; ↑ ACE and BUT (both positively correlated with *Ruminococcaceae_unclassified* and *Succinivibrio).* Similar PRO, and ↓ NH_4_^+^ (negatively correlated with *Ruminococcaceae_unclassified* and *Succinivibrio)* in all parts.
Míguez, 2020 [62]	6 elderly subjects (60–83 y)	POS mixtures (OGs 44.4%, AOS 16.9%, and GOS 11.6%)	Baseline and FOS from chicory (Sigma, Madrid, Spain)	6.5 g/d; pH 5.8; 0, 24, 48, and 72 h; TIM-2	Slower changes in composition compared with FOS over 72 h. Overall, beta diversity was closer to t0 h (i.e., greater) than FOS. Compared with baseline, ↓ Actinobacteria; ↑ P*revotellaceae* (similar to FOS), ↑ *Coprococcus, Phascolarctobacterium,* and *Prevotella,* and ↓ *Ruminococcus.* Compared with FOS: ↑ *Lachnospira, F. prausnitzii,* and ↓ *Lachnospiraceae_g* and *Ruminococcus.* ↑ total SCFA compared with FOS and baseline and ↑ ACE at 72 h.

Legend: Upward arrows indicate an increased bacterial abundance, and downward arrows indicate a decreased bacterial abundance. ^1^ Test product were described as in the studies, and they were produced under laboratory conditions when no supplier was mentioned. ^2^ All studies used a non-targeted bacterial group determination approach within their methodology unless specified. ^3^ AC: ascending colon; ACE: acetic acid; AOS: arabino-oligosaccharides; AXOS: arabino-xylo-oligosaccharides; BIOSTAT^®^: stirred-glass bioreactor; BMI: body mass index; BCFA: branched-chain fatty acids; BUT: butyric acid; DB: degree of branching; DC: distal colon; DE: degree of esterification; DM: degree of methylation; DP: degree of polymerization; FOS: fructo-oligosaccharides; Gal: galactose; GalA: galacturonic acid; GOS: galacto-oligosaccharides; LAC: lactate; MW: molecular weight; NH4^+^: ammonium; ND: not described; OGs: oligogalacturonides; PC: proximal colon; POS: pectic oligosaccharides; PRO: propionic acid; RG: rhamnogalacturonan; SBP: sugar beet pectin; SCFA: short-chain fatty acids; SD: significant differences; SHIME^®^: Simulator of Human Intestinal Microbial Ecosystem; SIEM: simulated ileal efflux medium; TC: transverse colon; TIM-2: TNO in vitro model of the colon; WC: waist circumference. ^4^ *Lachnospira eligens* previously known as *Eubacterium eligens* [23].

## 4. Discussion

To our knowledge, this is the first systematic review aiming to elucidate the prebiotic effect of pectic substrates investigated in vitro using human gut microbiota. Pectin and other pectin-derived substrates such as hydrolyzed pectins, RG1, and oligosaccharides have been widely studied, and recent studies even indicate that many of the structural characteristics of pectin have been shown to influence the utilization of these substrates by the gut microbiota. This systematic review demonstrates that fermentation of pectic substrates with human gut microbiota appears to have beneficial effects in terms of specific gut microbiota modulation and SCFA production.

### 4.1. Influence of the Methodology on the Fermentation of Pectic Substrates

Simple fermentation models such as batch fermentations and more complex continuous fermenter models can capture the complex microbial interactions driven by dietary substrates [18]. As of today, no standardized in vitro methodology exists for studying the gut microbiome. Differences in the methodology of these fermentation systems can influence the microbial abundances and metabolite production. The main differences observed between the fermentation systems were pH control, DNA-based techniques for gut microbiota analysis, and fecal inoculum.

The pH conditions play a key role in the competition between bacteria from different phyla or families that share the ability to utilize the same substrate. In pectin fermentations, a slightly acidic pH (pH < 6) suppresses the growth of *Bacteroides* spp. [55]. Even though most batch fermentations and continuous fermenters can detect an increase in this genus with different pectic substrates regardless of the pH, the effect is more obvious in pH-controlled studies [4,38,39,40,42,43,49,54,55,56]. Furthermore, growth inhibition of *F. prausnitzii* in fermentations with apple pectin at a mildly acidic pH has been reported to be strain-dependent [63]. However, the effect on this species in fermentations of pectic substrates has been detected regardless of pH control in both batch [34,39] and continuous fermenters [4,55]. 

In terms of differences in the DNA-based techniques for gut microbiota analysis used in the studies, most studies using a non-targeted approach detected an increased abundance of the genus *Lachnospira* [9,24,29,51,52,53,58,59,62]. The fact that the genus *Lachnospira* was not investigated in studies targeting specific bacterial groups could be a possible explanation for its non-detection in these studies. On the contrary, studies using a targeted-approach detected the increased abundances of the genus *Lactobacillus* and the *Lactobacillus-Enterococcus* group [32,33,34,35,40,43,46,57], as well as of *C. histolyticum* [32,34,35,40,42]. Changes in *Bifidobacterium* abundances were observed in most studies regardless of the DNA-based technique used for gut microbiota analysis. However, this finding is more obvious in studies using a targeted approach, suggesting that this method might be more accurate for detecting the change in this genus [4,31,32,33,34,35,39,40,41,42,43,46,57].

Using a pooled fecal inoculum rather than the microbiota from a single individual remains controversial, particularly with the concern of how representative such an inoculum is in regard to the colonic ecosystem, considering the abundance and the variety of bacterial species [17,64]. Overall, no major differences were observed between the studies performed with a fecal inocula from single donors compared with pooling. One study recently compared both ways of preparing a fecal inoculum (pooled and single) in an in vitro system monitoring the composition and activity of the gut microbiota under a standard TIM-2 fermentation [17]. Despite some differences observed in certain groups of bacteria in the single-donor fermentations, no major differences were obtained in terms of diversity in the gut microbiota. The majority of the operational taxonomic units (OTUs) were shared among both types of inocula (e.g., *Oscillibacter*, *Alistipes*, *Bacteroides* and *Dorea* genera were detected to have similar levels under both conditions). Furthermore, a similar metabolic activity in terms of SCFA production in the experiments using fecal inocula prepared from individuals and a pool of these was observed, indicating that the metabolic response of the gut microbiota present in both types of inocula was similar under both conditions.

### 4.2. Common Features for Pectic Substrates in Terms of Fermentation Rate, Gut Microbiota Composition, and SCFA Production

In comparison with other fibers and prebiotics, (e.g., fructans and galacto-oligosaccharides), microbiota fermentation of pectic substrates is slower (within 18-30h) [38,44,51]. The gradual fermentation is also observed when comparing pectic oligosaccharides (POS) to other oligosaccharides, such as fructo-oligosaccharides (FOS) with a similar degree of polymerization (DP) [41]. This phenomenon may be accentuated when pectic substrates are fed to the human gut microbiota in mixtures with other fibers (which may resemble the common human diet) rather than individually [29]. The available literature in humans confirm the slow and complete fermentation of pectic substrates in the large intestine [5], being non-detectable in the feces of adults during intervention studies even at daily doses of up to 30–40 g [65]. Gas production during fermentation (including breath hydrogen, H_2_) is commonly lower with pectin than with other fibers (e.g., wheat bran, fructans, and lactulose) in both in vitro [26] and human clinical studies [66,67]. Commonly used rapidly fermented prebiotics may lead to discomfort and flatulence in humans, especially at higher doses [68]. In contrast, non-digestible carbohydrates that are slowly fermented may reach the distal part of the colon and promote the growth of beneficial gut microflora, resulting in beneficial metabolites involved in the prevention of intestinal or metabolic diseases [41,69].

#### 4.2.1. The Effects of Pectic Substrates on the Gut Microbiota Composition

Despite slower fermentation, due to the complex molecular structure of pectic substrates, drastic changes to the gut microbiota composition can be induced which are stronger than FOS or type-2 resistant starch for instance [1,24]. These changes are not observed at the phylum or family level (except for *Ruminococcaceae*), but rather at the genus, species, and strain level [49,55]. Bacteroidetes, commonly regarded as dominant plant polysaccharides degraders in the human gastrointestinal tract [70], are decreased in some in vitro fermentations with pectic substrates [27,36,46,47], and they increased in three studies using different RGI fractions [25,31,51]. Among Bacteroidetes, the *Bacteroidaceae* family is generally equipped with several enzymes (40–50) able to degrade pectin (e.g., polysaccharide lyases, glycoside hydrolases, and carbohydrates esterases) [1,71]. Utilization of pectic substrates, and pectin in particular, is relatively common among *Bacteroides* spp., which seem to respond rapidly and specifically to the presence of polysaccharides in their environment [72]. In particular, *B. vulgatus* [49,54], *B. dorei* [31,54,56], and *B. stercoris* [54,55] are significantly stimulated during in vitro fermentation, especially at more neutral pH levels such as those in the descending colon [49,55].

The phylum Firmicutes is variably influenced by pectin during in vitro fermentation. Within this phylum, the family *Ruminococcaceae*, mostly promoted by pectin in continuous fermenters, represents abundant members of the normal microbiome, reaching approximately 10–20% of abundance in healthy humans, where they break down indigestible carbohydrates and produce SCFA [73]. *F. prausnitzii*, a member of the *Ruminococcaceae* family, is one of the dominant butyrate producers in the human gut, with a prevalence of approx. 99% [74]. However, its relative abundance is reduced in case of ulcerative colitis or inflammatory bowel disease [75,76], and metabolic disorders [74,77]. Interestingly, *F. prausnitzii* is promoted in pectin fermentations with fecal samples from healthy subjects [34,39], but also with citrus pectin rich in RGI [27], RGII [39], as well as with oligosaccharides from citrus [34] or sugar beet [52]. In contrast, one study found that the abundance of *F. prausnitzii* varied in pectin fermentations based on its structural differences [59]. A plausible reason for these differences could be that substrate utilization by this species may be strain dependent, as previously reported [63]. The authors also confirm the ability of intestinal isolates of *F. prausnitzii* to utilize pectin from apple (but not citrus) in culture growth experiments and that it possesses small repertoires of carbohydrate-active enzymes (CAZyme) encoding genes involved in pectin degradation. 

The *Lachnospiraceae* family presents enzymatic capabilities to degrade pectic substrates among its members [71]. Particularly, *L. eligens* possesses various enzymes able to degrade pectin, at least two glycoside hydrolases, two polysaccharide lyases, and two carbohydrate esterases. These enzymatic capabilities are also represented in *Lachnospira* spp. and *Roseburia intestinalis*, but are absent in the genera *Coprococcus* and *Anaerostipes*. This variation in enzymatic capabilities to degrade pectin could justify why the modulation of the *Lachnospiraceae* family varied among studies. A significant increase in the relative abundance of the genus *Lachnospira* in the gut microbiota of healthy subjects was observed with citrus pectic substrates and various DM [24,28,59], polygalacturonic acid from citrus pectin [29], RGI [51], as well as potato fiber (rich in pectin) [58]. Additionally, *L. eligens* can also be stimulated by apple pectin (with no clear effect of a pH level between 5.5 and 6.5–6.9) [39,55]. In these studies, the specific stimulation of *L. eligens* or *Lachnospira* was quite unique to pectic substrates and not observed with fructans (FOS or inulin) or resistant starch [24,58]. *Lachnospira* is not stimulated in microbiota from obese subjects after fermentation with citrus pectin, possibly due to its very low initial concentration [61]. Interestingly, one study demonstrated that the effect is less visible when polygalacturonic acid is fed to the gut microbiota within a blend of three or six fibers, than when presented alone [29]. 

The selective modulation of these bacterial groups by pectic substrates can be beneficial. For example, the ability of pectin to stimulate *L. eligens* could be particularly important from a health perspective, since this bacterium is known to exert strong anti-inflammatory effects in vitro to an even greater extent than *F. prausnitzii* [71]. *L. eligens*, which is around 90–92% prevalent with a relative abundance between 1–2% [74,78], was recently identified as one of the top 30 bacterial species strongly correlated with the healthy eating index [74], or the Mediterranean diet [79], similar to *F. prausnitzii*. In these studies, the *L. eligens* species was particularly negatively correlated with visceral fat, blood triglycerides, and very low-density lipoprotein (VLDL) at 6 h but also with fasting and post-prandial glycoprotein acetylation, a biomarker of inflammation. In another clinical study conducted in type 2 diabetes patients, *L. eligens* was selectively increased by a high dietary fiber diet and negatively associated with postprandial glucose and insulin, body weight, and waist circumference [80].

#### 4.2.2. The Effects of Pectic Substrates on the Production of SCFA

The amount and type of fiber consumed can have dramatic effects on the composition of the intestinal microbiota, and consequently, on the type and amount of SCFA produced [81]. Overall, total SCFA production is generally moderate, but significant, during the fermentation of pectic substrates by the human gut microbiota. In the first hours of fermentation, the total SCFA levels were lower for pectic substrates compared with fructans. Furthermore, pectic substrates induce a larger amount of acetate produced by the gut microbiota from healthy adults and the elderly (60–83 years), regardless of their botanical source or structure when compared with other fibers [24,30,42,62]. One study showed that the SCFA levels that resulted from pectin fermentation (including acetate) were lower in the obese compared with the lean subjects [17]. Accordingly, two clinical studies showed higher acetate levels (in feces or blood) after dietary supplementation of 20–25 g of pectin, or citrus fiber containing pectin [82,83]. The large amounts of acetate commonly produced in the fermentations of pectic substrates may be in alignment with studies showing an increase in the relative abundance of *Lachnospira*, since some species of *Lachnospira* are known to produce acetate [84], and its level is negatively correlated with propionate in two in vitro studies [24,58].

Although more variable, the propionate and butyrate levels are lower compared with the fructans [26,29,38]. This finding is particularly observed in butyrate production in fermentations of different pectic substrates [26,34], hydrolyzed pectins and oligomers [40]. 

In terms of other metabolites, a limited amount of lactate was observed in pectin fermentations aligned with a limited increase of *Bifidobacterium* and *Lactobacillus,* which suggests that it could be further used by other bacteria via cross-feeding interactions [31,34,35]. Only five studies measured ammonia (NH_4_^+^) production during pectin fermentations, and its levels were either reduced [4,60,61] or unchanged [17,31].

#### 4.2.3. The Impact on Fermentative Activities Based on Donor Health Status

The effect of pectic substrates on the gut microbiota of overweight or obese subjects [17,49,61] is slightly different compared with healthy or normal weight subjects. For instance, *B. vulgatus* was stimulated by apple pectin and arabinogalactan only in the gut microbiota of normal weight children but not in overweight ones [49]. An increase in the relative abundances of *Bacteroides* spp., *Bifidobacterium*, *Catenibacterium*, *Clostridium* cluster XIVb, and *Parasutterella* were only observed with the gut microbiota from lean subjects with apple fiber and/or sugar beet pectin [17]. The authors argue whether the gut microbiota from overweight subjects might have lost their capacity to degrade the pectic structures due to depletion or a decrease in some bacterial species (e.g., *B. vulgatus* is reduced in overweight children compared with normal weight children) [49]. Interestingly, LM pectin promotes an increase in bacteria with potential anti-inflammatory effects (*Succinivibrionaceae* members) and SCFA levels, and a decrease in *Lachnospiraceae* in obese microbiota [61].

Two studies compared the effect of pectic substrates on the human gut microbiota from UC patients (in remission or relapse) with healthy adults [46,47]. In both studies, a treatment with arabino-oligosaccharides from sugar beet (DP 2–10) resulted in no significant differences in gut microbiota composition between the populations. However, slightly different effects were found between the studies. While both showed a reduction of Bacteroidetes, one study reported an increased relative abundance of Firmicutes (for oligosaccharides with a DP4 only) [47], and the other one reported a decrease in the same phylum [46]. Interestingly, a limited SCFA production was obtained in comparison with other studies, and the common production of acetate was only seen with microbiota from relapse subjects [46,47].

### 4.3. Structure-Function Relationship of Pectic Substrates

#### 4.3.1. Degree of Methyl-Esterification

The effect of DM of pectic substrates on the modulation of the gut microbiota was investigated in fives studies [27,30,32,40,59]. LM pectins were previously reported to be fermented faster than HM pectins [85]. Similar findings were observed in two studies comparing pectins with different botanical origins, production processes and molecular weights [27,30]. In contrast, HM pectins can induce a slightly higher production of SCFA, especially propionate [59]. Other authors also reported that DM does not influence the fermentability of pectic oligosaccharides or hydrolyzed pectin, as reflected by a similar SCFA yield and profile [40]. These contradictory findings may suggest that DM either has an overall moderate effect on gut microbiota modulation or that it might depend on the initial gut microbiota composition. The species *Prevotella copri*, and *F. prausnitzii* and the family *Ruminococcaceae* are positively correlated with DM, contrary to many genera such as *Coprococcus*, *Oscillospira*, *Prevotella*, *Ruminococcus*, and *Lachnospira* [59]. Regardless of whether these bacteria are considered fast or slow growers, they can impact fermentation kinetics and induce different results between LM and HM pectins in terms of the rate of SCFA production.

#### 4.3.2. Composition of Neutral Sugars

The nature of neutral sugars and the types of linkages in the pectin structure may influence the composition and activity of the human gut microbiota. In this regard, gluco-oligosaccharides and galacto-oligosaccharides are fermented faster than arabino-oligosaccharides, and even faster than oligogalacturonides, for which there is a lag time of 5–7 h in vitro [32,34,35]. Galactan molecules are degraded by glycoside hydrolases (GHs). For example, GH2 is present in the genome of many genera in the human gut, such as *Bacteroides*, *Bifidobacterium*, and *Ruminococcus*, and species such as *F. prausnitzii*, *R. intestinalis*, *Akkermansia muciniphila* and *Escherichia coli*. The genera *Bifidobacterium* and *Lactobacillus* possess enzymatic capabilities to degrade β-1,4-galactan chains as a carbon source in potato pulp [37]. The widespread presence of these enzymes in the gut commensals may explain why these molecules are quickly fermented. Furthermore, enzymes such as the GH families that can degrade arabinans (e.g., GH51, GH43, GH27, and GH127), are also largely present in *Bacteroides* spp. and several species of *Bifidobacterium* (e.g., *B. longum subsp*. *longum* or *B. adolescentis*). On the contrary, enzymes such as glycoside hydrolases and polysaccharide lyases (PLs) that can degrade the RGI backbone (e.g., PL11) are only present in some *Bacteroides* spp., and only the GH type appears to be present in *F. prausnitzii*, *R. intestinalis*, *Klebsiella oxytoca*, *Enterobacter cloacae*, and *A. muciniphila* [1].

*Bifidobacterium* spp. (at least *B. adolescentis* and *B. breve*) show a poor capacity to degrade pectin or the RGI backbone contrary to *Bacteroides* [71], but it seems to be more adapted to degrading side chains made of arabinan and galactan [1,37]. This is in agreement with a pure culture experiment showing that *Bifidobacterium* spp. can partially metabolize distinct types of apple pectin-derived oligosaccharides, except oligogalacturonides [35]. Similarly, *Bifidobacterium* exhibits a preference for oligosaccharides rich in galactose and arabinose, as shown by several in vitro studies [33,42,43]. *Bifidobacterium* further develops with alkali-extracted pectin containing the highest RGI ratio (≈60%) than with pectin obtained by acid extraction presenting a lower RGI ratio (≈26%) [27]. Previous genomic and in vitro studies have shown that *Bifidobacterium* preferentially utilizes arabinan (sugar beet) and arabinogalactan (potato) rather than polymers or oligomers of GalA [42]. Finally, this genus also increases in fermentations with sugar beet arabino-oligosaccharides [33,36,41], and apple-derived oligosaccharides [35]. This preference for RGI certainly because of its side chains has been confirmed in humans, where a dietary supplementation with 15 g of potato fiber did not induce a significant modification of fecal microbiota. However, RGI derived from the same fiber could increase the relative abundance of *Bifidobacterium* [86]. Additionally, in infants, the consumption of formulae containing citrus pectin-derived oligosaccharides could stimulate the growth of *Bifidobacterium* [87].

The presence of arabinose (and arabinan side chains) also seems to be positively correlated with higher counts of *Prevotella*, (and *P. copri* in particular) but negatively correlated with *Lachnospira*, *Bacteroides ovatus*, and members of *Coprococcus* [59]. In most studies, *Prevotella* is investigated jointly with other bacterial genera such as *Bacteroides*, but not on its own, thus not allowing the confirmation of a strong effect of pectic substrates on its growth. Furthermore, the low abundance of *Prevotella* could also be due to the lower degree of branching and/or arabinose content of the pectic substrates tested in these studies compared which RGI and sugar beet pectin. Since *P. copri* is generally associated with high plant fiber diets and favorable postprandial glucose metabolism [74], further studies are needed to provide evidence of its fermentative activities. 

Interestingly, these structural differences in terms of the neutral sugar composition of pectic substrates, as described above, may result in different fermentation patterns and activities by the gut bacteria. In particular, galactan and arabinan and their respective oligosaccharides, may promote higher total SCFA levels compared with fermentations with polygalacturonic acid and pectin [42]. 

#### 4.3.3. Distribution of HG and RG Fractions

The distributions of the linear and branched structural regions of pectin have been shown to influence its fermentation by the human gut microbiota. The *Ruminococcaceae* family and especially *F. prausnitzii* positively correlated with GalA and DM [4,17,59]. This suggests that different microorganisms may exhibit specific preferences for defined substrates, which may be the case for *F. prausnitzii* that preferentially utilized the HG backbone compared with RG, and HM compared with LM pectins. Previous research has demonstrated the enzymatic capabilities of *F. prausnitzii* to degrade pectin and RGI-backbone [1], as well as GalA [88]. The utilization of the latter in particular was also confirmed for several strains of *F. prausnitzii* [63]. These strains are able to compete with *B. thetaiotaomicron* and *L. eligens* for pectin degradation, especially in mildly acidic pH conditions. Interestingly, the ability of the specific strains of *F. prausnitzii* to function both as a consumer and a producer of acetate, depending on the conditions, has been previously reported, and in particular, a higher uptake of GalA as carbon source is facilitated by acetate production by the bacteria [88].

#### 4.3.4. Degree of Branching

The degree and distribution of neutral sugar branches attached to the RGI region may influence the molecular conformation of pectins [89], and further influence their fermentability [42]. Pectins derived from sugar beet or apple are usually characterized by a higher degree of branching compared with those of a citrus origin [89]. Sugar beet-derived pectin and POS may be fermented more slowly, and not completely, than from apple or citrus [32]. Furthermore, the genus *Lachnospira*, particularly promoted in pectin fermentations, is positively correlated with the degree of branching [59]. The same study also showed a similar finding regarding the genus *Coproccocus*, contrary to the species *P. copri*.

#### 4.3.5. Molecular Weight

The molecular structure, and even nature of monosaccharide units, may influence the kinetics and extent of SCFA production. POS are generally fermented faster and to a greater extent (resulting in the greater production of SCFA and pH reduction) than pectin polysaccharides from the same botanical origin [32,34,42]. A minor difference in the degree of polymerization (four vs. five) from the same type of substrate induces a different effect on the gut microbiota. While a decrease in the Bacteroidetes phylum was observed for both oligosaccharides, a significant increase in Firmicutes was only seen with the substrate with DP4 [47]. Furthermore, *Bifidobacterium* was more stimulated by oligosaccharides from potato-derived RG1 (without a difference between DP 2–12 and DP 13–70) than by polysaccharides [57]. A similar effect was observed in fermentations with modified pectins from citrus and artichoke compared with their parent pectins [43]. The genus *Bifidobacterium* exhibits a well-known preference for shorter molecules (e.g., the fermentation of fructan-derived substrates), as was recently reviewed [90], even though this was not observed in a previous study with different pectic substrates [42]. In contrast to *Bifidobacterium*, the effect of the DP on *F. prausnitzii* may be strain-dependent. Certain *F. prausnitzii* strains possess some ability to utilize apple pectin, and in common with *L. eligens*, they can utilize the galacturonide oligosaccharides with DP4 and DP5 derived from sugar beet pectin [71].

#### 4.3.6. Other Structural Characteristics

One study investigated the possible impact of the presence of amide groups in the pectin structure on the gut microbiota [59]. The authors reported that amidated citrus pectins had a similar effect on the gut microbiota in comparison to non-amidated pectins from citrus or sugar beet. However, both pectins shared a similar (low) DM, and this parameter might have had a stronger influence on the modulation of the gut microbiota than amidation. Another study investigated the effect of feruloyl substitution of short (DP 2–10) and long-chain (DP 7–14) arabino-oligosaccharides on the gut microbiota [36]. In this study, *Bifidobacterium* was similarly and selectively stimulated by both the feruloylated and non-feruloylated arabino-oligosaccharides. The potential specific effect of this structural difference on the gut microbiota may have been hidden by a greater effect of different DPs between the substrates.

## 5. Conclusions

Based on this comprehensive review, we found evidence to support the potential prebiotic effect of pectic substrates based on in vitro studies, as shown by a specific fermentation profile at the genus, species, and even strain levels. It is evident that most pectic substrates can stimulate the growth of *Bacteroides* and *Lachnospira* genera as well as species such as *F. prausnitzii* and *L. eligens*, and increase the production of SCFA (acetate in particular). The structural characteristics of pectic substrates have been shown to influence their utilization by the gut microbiota. *Bifidobacterium* in particular, shows a preference for the fermentation of shorter molecules, especially arabinose-rich side chains such as from RGI. *F. prausnitzii* prefers HG to RG, but its preferences are less clearly defined and might be determined at the strain rather than species level. 

Even though these findings are promising, they should be carefully interpreted since direct extrapolation to humans cannot be performed. A recent clinical trial where a dietary intervention of 15 g/d of sugar beet pectin for 4 weeks resulted in no significant changes in gut microbiota composition or SCFA production [15]. Furthermore, the fermentation profile of pectic substrates by the human gut microbiota varies depending on whether the fiber is provided alone or in a blend, which is more representative of the typical human diet, as was previously reported [29]. Therefore, well-designed clinical trials are needed to prove the specific effect of these substrates on human gut microbiota and the potential health effects derived from it.

## Figures and Tables

**Table 1:** Description of the various structures of pectic substrates used in the studies.

Pectic Substrates	Origin	Molecular Structure and Main Linkages	Other Parameters Described in the Studies
Pectin	Citrus, apple, sugar beet, soy, sunflower, artichoke, and prune	(GalA)_n_ and/or (GalA-Rha)_n_ and/or (GalA-Gal)_n_; α(1,4); α(1,2)	GalA: 32–88% DE: 2–79%
Hydrolyzed pectin	Citrus, sunflower, and artichoke		GalA: 56–79% DE: 5–17% MW: 9.2–300 kDa
OS from pectin	Methylated citrus pectin, orange or lemon peel, and apple		GalA: 42–96%; DE: 29–62%; DP 1–10 or MW > 23 kDa
	Sugar beet		GalA: <2–78%; Ara: 10–85%; DP 2–10 or MW: <1–12 kDa
Polygalacturonic acid	Citrus pectin	α(1,4)GalA_n_	GalA: >90%
OS from PolyGalA	Polygalacturonic acid		GalA: 91–98% DP 1–23
RG1- enriched	Okra, carrot, *A. thaliana* seed mucilage, prune, lime, and potato	α-(1,2)(Rha)_n_ and α-(1,4)(GalA)_n_ and β-(1,4)(Gal)_n_ (potato only), and α-Ara and β-D-Gal residues of different sizes	GalA: 10–25%; Ara > 48%; Potato: Gal 61%, 34 kDa
OS from RG1	*A. thaliana* seed mucilage; Potato		Potato: >70% Gal; DP 2–70
Arabinan	Sugar beet	α-(1,5)(Ara)_n_ and possible Ara residues or short side chains	MW: 18 kDa, debranched, Ara:Gal:Rha = 71:26:3
OS from Arabinan			DP 1–11, Ara: 93.4%
Arabinogalactan	Acacia fiber and larch tree	AGI: β-(1,4)-D-(Gal)_n_ and occasional β-(1,3), and α-Ara/Fuc/GlucA AGII: β-(1,3)-D-(Gal)_n_ and β-(1,6)-D-Gal/Ara	MW: 300–800 kDa
Galactan	Potato	β-(1,4)(Gal)_n_ and may contain Ara/Rha/GalA	MW: ~100 kDa
OS from Galactan			Gal: 95%, DP 1–10
Galactomannan	Carob tree and guar plant	Man(β-1,4)[Gal(α-1,6)]β-Man	MW: 1.07 × 10^5^–0.67 × 10^6^ kDa
Fibers rich in pectin	Potato	α-(1,2)(Rha)_n_ and α-(1,4)(GalA)_n_ and β-(1,4)(Gal)_n_ side chains	65% fiber; GalA: 13%
	Chicory root pulp	Pectin fraction: (GalA)_n_ and/or (GalA-Rha)_n_; α(1,4); α(1,2). Inulin fraction: β-(2-1)(Fru)_n_	62% pectin, uronic acids 38%
	Apple	α-(1,2)(Rha)_n_ and α-(1,4)(GalA)_n_ and α-(1,4)(Ara)_n_, β-(1,4)(Gal)_n_	GalA: 23%, 60% total sugars (45% glucose)
	Citrus fiber	(GalA)_n_ and/or (GalA-Rha)_n_; α(1,4); α(1,2)	42% pectin, 25% cellulose and hemicellulose

Legend: Ara: arabinose; AG: arabinogalactan; AOS: arabino-oligosaccharides; DE: degree of methyl esterification; DP: degree of polymerization; Fuc: fucose; Fru: fructose; Gal: galactose; GalA: galacturonic acid; GlucA: glucuronic acid; GOS: galactooligosaccharides; HG: homogalacturonan; Man: mannose; MW: molecular weight; RG: rhamnogalacturonan; Rha: rhamnose.

## Supplementary Materials

The following supporting information can be downloaded at: https://www.mdpi.com/article/10.3390/nu14173629/s1, Table S1: The search strategy used for identification of the relevant studies included in the systematic review.
