# Supplementary material for: The Potential of Pectins to Modulate the Human Gut Microbiota Evaluated by In Vitro Fermentation: A Systematic Review
Source: Nutrients. 2022 Sep 2;14(17):3629. doi: 10.3390/nu14173629 (PMC9460662; doi:10.3390/nu14173629)
Supplement: Supplementary file 1 [file nutrients-14-03629-s001.zip › nutrients-1861907-supplementary.pdf]

nutrients MDPI

Supplementary Materials

The Potential of Pectins to Modulate the Human Gut Microbiota Evaluated by In Vitro Fermentation: A Systematic Review

Supplementary Table S1. The search strategy used for identification of the relevant studies included in the systematic review can be seen below.

Database	Search strategy
PubMed	((pecti*[Title/Abstract]) OR (galacturo*[Title/Abstract]) OR (rhamnogalacturo*[Title/Abstract]) OR (polygalacturo*[Title/Abstract]) OR (homogalacturo*[Title/Abstract]) OR (pectate[Title/Abstract]) OR (arabino-oligosaccharide*[Title/Abstract]) OR (arabinan[Title/Abstract]) OR (pAOS[Title/Abstract]) OR ("cane fiber"[Title/Abstract]) OR ("beet fiber"[Title/Abstract]) OR ("citrus peel fiber"[Title/Abstract]) OR ("citrus fiber"[Title/Abstract]) OR ("apple fiber"[Title/Abstract])) AND ((prebiotic*[Title/Abstract]) OR (microbi*[Title/Abstract]) OR (flora[Title/Abstract]) OR (short chain fatty acid*[Title/Abstract]) OR (short-chain fatty acid*[Title/Abstract]) OR (scfa[Title/Abstract]) OR (butyrate[Title/Abstract]) OR (acetate[Title/Abstract]) OR (propionate[Title/Abstract]) OR (lactate[Title/Abstract]) OR (ferment*[Title/Abstract]) OR (intestin*[Title/Abstract]) OR (gastro*[Title/Abstract]) OR (fecal[Title/Abstract]) OR (faecal[Title/Abstract]) OR (feces[Title/Abstract]) OR (faeces[Title/Abstract]) OR (stool*[Title/Abstract]) OR (butyrogenic[Title/Abstract]) OR (bifidogenic[Title/Abstract])) AND ((vitro[Title/Abstract]) OR (donor*[Title/Abstract]) OR (human*[Title/Abstract]) OR (patient*[Title/Abstract]) OR (adult*[Title/Abstract]) OR (child*[Title/Abstract]) OR (infant*[Title/Abstract]) OR (elderly[Title/Abstract]) OR (senescence[Title/Abstract]) OR (bifido*[Title/Abstract]) OR (lactobacill*[Title/Abstract]) OR (bacteroide*[Title/Abstract]) OR (enterococc*[Title/Abstract]) OR (faecalibacterium[Title/Abstract]) OR (roseburia[Title/Abstract]) OR (clostridi*[Title/Abstract]) OR (ruminococc*[Title/Abstract]) OR (coprococc*[Title/Abstract]) OR (eubacterium[Title/Abstract]) OR (lachnospira*[Title/Abstract]) OR (Akkermansia[Title/Abstract]) OR (probiotic*[Title/Abstract]) OR (firmicutes[Title/Abstract]) OR (subject*[Title/Abstract]) OR (volunteer*[Title/Abstract]))
Scopus	TITLE-ABS (pecti* OR galacturo* OR rhamnogalacturo* OR polygalacturo* OR homogalacturo* OR pectate OR arabino-oligosaccharide OR arabinan OR (pAOS) OR "cane fiber" OR "beet fiber" OR (citrus PRE/1 fiber) OR "apple fiber") AND TITLE-ABS (prebiotic* OR microbi* OR flora OR "short chain fatty acid" OR scfa OR butyrate OR acetate OR propionate OR lactate OR ferment* OR intestin* OR gastro* OR fecal OR faecal OR feces OR faeces OR stool OR butyrogenic OR bifidogenic) AND TITLE-ABS (vitro OR donor OR human* OR patient OR adult* OR child* OR infant* OR elderly OR senescence OR bifido* OR lactobacill* OR bacteroide* OR enterococc* OR faecalibacterium OR roseburia OR clostridi* OR ruminococc* OR coprococc* OR eubacterium OR lachnospira* OR Akkermansia OR probiotic* OR firmicutes OR subject OR volunteer)
